# Prognostic model for oversurvival and tumor-specific survival prediction in patients with advanced extrahepatic cholangiocarcinoma: a population-based analysis

**DOI:** 10.1186/s12876-023-03017-6

**Published:** 2023-11-30

**Authors:** Yu Zhang, Chunzhong Qiao, Peng Zhao, Changhe Zhang

**Affiliations:** 1 Postgraduate School, Dalian Medical University, Dalian, China; 2 Department of General Surgery, The Affiliated Taizhou people’s Hospital of Nanjing Medical University, Taizhou, China

**Keywords:** Advanced ECCA, Nomogram, Oversurvival, Cancer-specific survival

## Abstract

**Background:**

The prognosis of patients with extrahepatic cholangiocarcinoma (ECCA) must be determined with precision. However, the usual TNM staging system has the drawback of ignoring age, adjuvant therapy, and gender and lacks the ability to more correctly predict patient prognosis. Therefore, we determine the risk factors of survival for patients with advanced ECCA patients and developed brand-new nomograms to forecast patients with advanced ECCA’s overall survival (OS) and cancer-specific survival (CSS).

**Method:**

From the Epidemiology and End Results (SEER) database, patients with advanced ECCA were chosen and randomly assigned in a ratio of 6:4 to the training and validation subgroups. The cumulative incidence function (CIF) difference between groups was confirmed by applying Gray’s and Fine test and competing risk analyses. Next, the cancer-specific survival (CSS) and overall survival (OS) nomograms for advanced ECCA were developed and validated.

**Results:**

In accordance with the selection criteria, 403 patients with advanced ECCA were acquired from the SEER database and then split at random into two groups: a training group (*n* = 241) and a validation group (*n* = 162). The 1-, 2-, and 3-year cancer-specific mortality rates were 58.7, 74.2, and 78.0%, respectively, while the matching mortality rates for the competition were 10.0, 13.8, and 15.0%. Nomograms were generated for estimating OS and CSS, and they were assessed using the ROC curve and the C-index. The calibration curves showed that there was a fair amount of agreement between the expected and actual probabilities of OS and CSS. Additionally, greater areas under the ROC curve were seen in the newly developed nomograms for OS and CSS when compared to the 7th AJCC staging system. The advanced ECCA patients were divided into groupings with an elevated risk and those with a low risk and the Kaplan-Meier method was used for the survival analysis, which showed that survival time was shorter in the high-risk group than in the low-risk group.

**Conclusion:**

The proposed nomograms have good predictive ability. The nomograms may can help doctors determine the prognosis of patients with advanced ECCA as well as provide more precise treatment plans for them.

**Supplementary Information:**

The online version contains supplementary material available at 10.1186/s12876-023-03017-6.

## Introduction

Cholangiocarcinoma (CCA) is the second most widespread malignant tumor after hepatocellular carcinoma (HCC) [1], and it is an extremely deadly tumor that develops from the bile duct epithelium. The anatomical division of CCA include intrahepatic cholangiocarcinoma (ICCA) and extrahepatic cholangiocarcinoma (ECCA) with the latter accounting for roughly 70–90% and the former for merely 10–20% of all CAA, respectively [2]. Nevertheless, the majority of patients were discovered at an advanced stage and missed the optimal opportunity to be treated [3] owing to the poor clinical presentation [4], the lack of evidence of identifiable biochemical indications, and the high level of aggressiveness. Hence, the prognosis for ECCA is often poor [5, 6], with a 5-year survival rate of 11–31% [7]. The sole curative approach for ECCA is surgery and about one-third of patients are candidates for surgery.

Additionally, much advanced ECCA patients have a combination of several comorbidities, such as hypertension, diabetes, and heart disease, and the mortality from these diseases increases with age [8–10]. Therefore, these risk factors need to be taken into account when assessing prognosis, however, the presence of competing risks has not been taken into account in previous studies, biasing the conclusions obtained [11, 12]. Hence, competing risk factors should be included in the analysis when assessing the prognosis more accurately.

The prognosis of patients with ECCA is frequently assessed through the American Joint Committee on Cancer (AJCC) Tumor-Node-Metastasis (TNM) [13] approach, although this system solely assesses tumor features without taking other clinical features into account such as gender, age, and adjuvant therapy. It is not effective in predicting the overall prognosis of ECCA patients, therefore, a more comprehensive and effective staging system is warranted to predict the prognosis of patients with advanced ECCA. Nowadays, it is common practice to forecast the prognosis of a wide range of diseases utilizing clinically based nomograms, which incorporate all risk variables into a thorough analysis [14–17]. In addition to making clinical forecasts and better individualized treatment plans, it is more intuitive and can aid physicians.

According to the 7th AJCC-TNM stage used in this investigation, we classified the patients with stage IIIA to IVB as having advanced ECCA. The competing risk factors were assessed, and appropriate nomograms were developed based on data screened from the Surveillance, Epidemiology, and End Results (SEER) database to investigate the overall survival (OS) and cancer-specific survival (CSS) of patients with advanced ECCA.

## Materials and methods

### Patients

A retrospective examination of patients who had an advanced ECCA diagnosis during 2000–2020 was performed in the SEER database. Data on cancer consequences is available in the database from 18 cancer registries, which account for 30% of the US population [18]. Since the SEER database is accessible to the general public and does not include any data that could be used to identify patients, institutional ethical approval and informed permission are not required. The following conditions must be met: 1. ECCA diagnosis 2. TNM stage identified as advanced 3. full clinical and pathological data. The following are the exclusion requirements: 1. no confirmed diagnosis 2. insufficient clinical or pathological data 3. incomplete follow-up informations 4. individuals having an early ECCA diagnosis. The flow chart in Fig. S4 shows the process of screening. The trial included 403 individuals with the aforementioned illnesses in total, then these patients were randomly divided into the training and validation groups (6:4).

### Data collection

Patients’ clinical and pathological data, such as gender, age, race, TNM stage, grade, method of diagnosis, year of diagnosis, surgery, radiation, chemotherapy, follow-up data, and reason of death, were gathered and evaluated. The 7th AJCC staging was used to stage the tumors in this study, and it included two event endpoints: OS, which was defined as the period from the time of diagnosis up to the time of one’s final follow-up visit or death from whatever reason occurred, and CSS, which was defined as the time from the diagnosis to the date of cancer death [19], excluding deaths from other causes. In addition, patients who are lost to follow-up are not included.

### Statistical analysis

With regard to baseline characteristics, categorical variables were compared between the training and validation groups using the chi-quared test or Fisher’s exact test. Variables were depicted as frequencies and proportions, while survival times were shown as median and interquartile range (IQR) values. Fatalities related to cancer and fatalities not related to cancer were viewed as competing events. The cumulative incidence function (CIF) difference between groups was confirmed using the Fine and Gray’s test. The OS was analyzed using the Kaplan-Meier method, and the log-rank test was performed to assess survival disparities. Univariate analysis was used to identify prognostic factors that had a substantial impact on OS and CSS, and positive factors (*p*<0.05) were then subjected to multifactorial analysis. Then nomograms were created using the identified parameters to forecast the prognoses of advanced ECCA patients at 1-, 2-, and 3-year. The receiver operating characteristic curve (ROC) and concordance index (C-index) were used to rate the nomograms’ capacity for judgment [20]. To compare the real with anticipated values, calibration curves were created. And for further contrasting the accuracy of the nomograms and the 7th AJCC staging, the integrated discriminant improvement (IDI) and net reclassification improvement (NRI), respectively, were computed. Finally the advanced ECCA patients were divided into groupings with an elevated risk and those with a low risk based on their total score after the addition of the nomogram-based criteria. The log-rank test was used to assess the prognostic characteristics of the two groups, and the Kaplan-Meier method was used for the survival analysis.

In order to complete all statistical analyses, R software (version 4.1.2) was used. Statistics were considered significant if *p*<0.05.

## Results

### Personalities of patients

In accordance with the selection criteria, 403 patients with advanced ECCA were acquired from the SEER database and then split at random into two groups: a training group (*n* = 241) and a validation group (*n* = 162). Overall, 58.6% of patients were under 65 years old, 92.1% of patients were white, and 52.4% of patients were men. Less than half of the patients (48.6%) did not have lymph node metastasis(N0), and only 6.5% had farther lymph node metastasis (N2). Only 22.8% of patients underwent surgery, but there are 44.7 and 27.0% of patients underwent chemotherapy and radiotherapy, respectively. Patient demographics, clinicopathology, and features did not differ statistically significantly (Table S1).

**Fig. 1:**
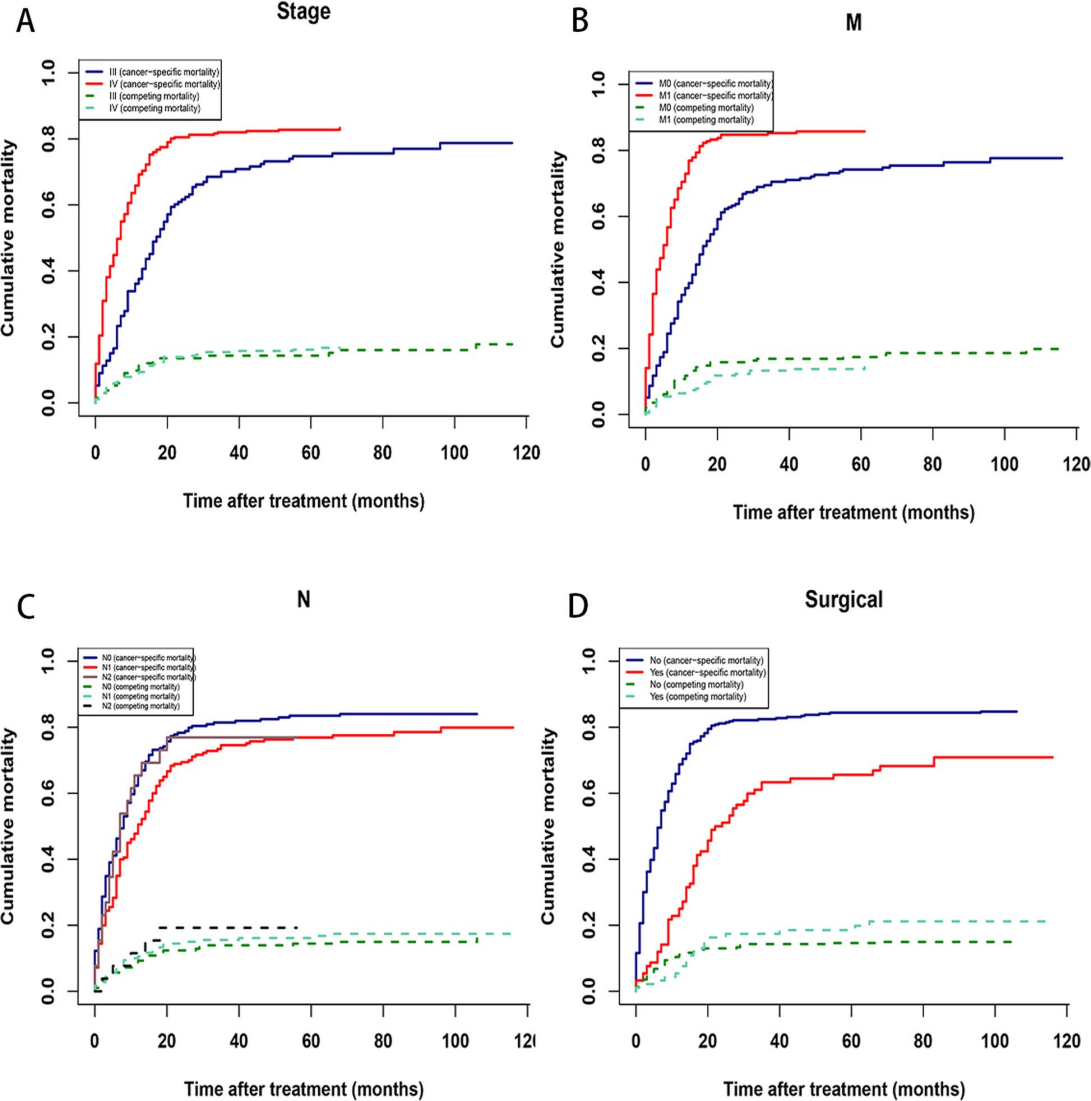
Cumulative cancer-specific and competing mortality stratified by patient characteristics: **A** stage; **B** M stage; **C** N stage; **D** surgical treatment

**Fig. 2:**
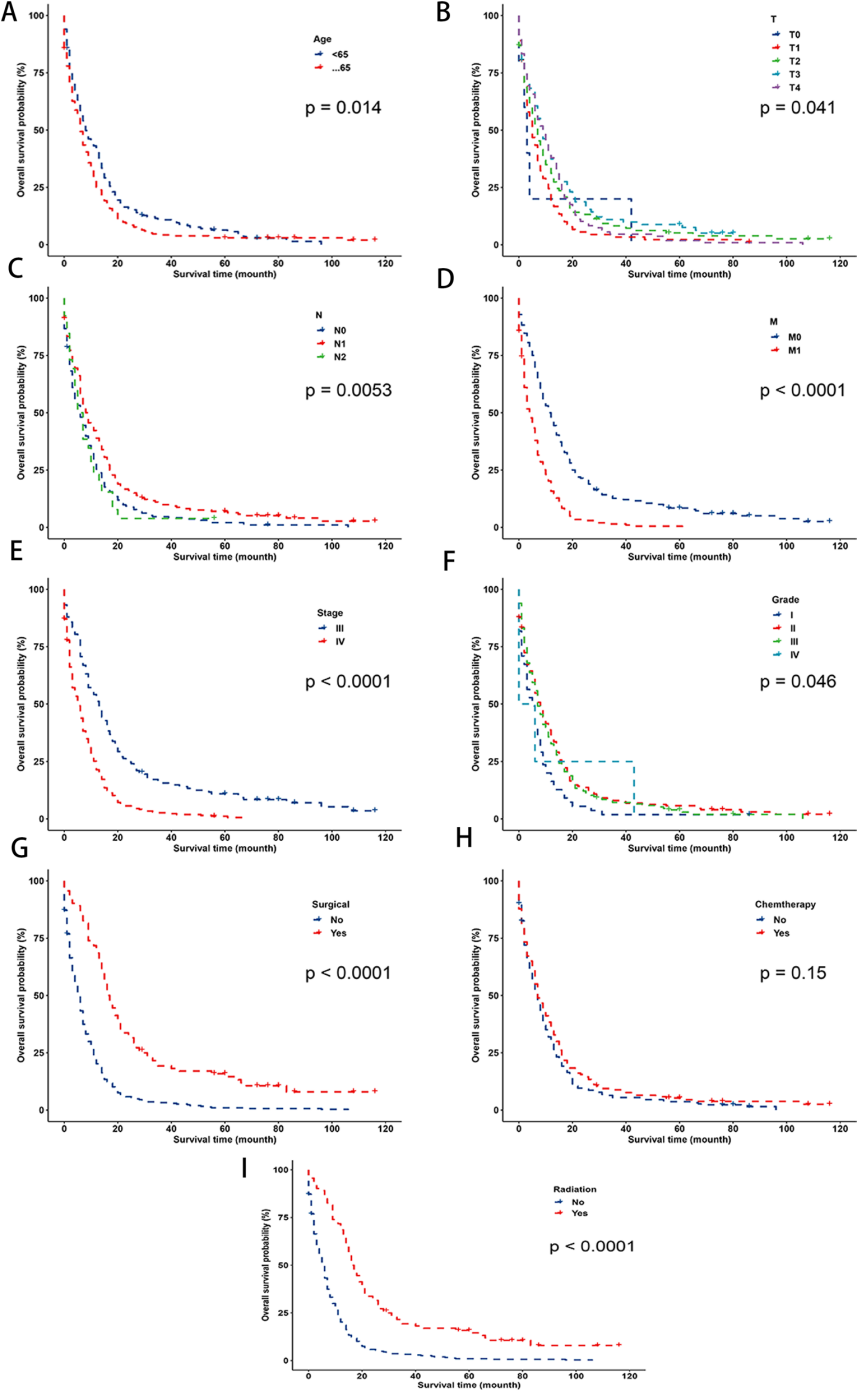
Overall survival rates stratified by patient characteristics: **A** age; **B** T stage; **C** N stage; **D** M stage; **E** TNM stage; **F** grade; **G** surgical treatment; **H** chemtherapy treatment; **F** radiation treatment

We recorded 390 deaths throughout the course of the 10-month follow-up period, including 324 deaths from cancer and 66 deaths from other causes. According to Table 1, the incidence of OS was 31.3, 12.0, and 7.0%, respectively, at 1-, 2-, and 3-year but the corresponding cumulative incidence of ECCA fatalities was 58.7, 74.2, and 78.0%. Distant metastases, TNM stage, T stage, and surgical intervention were identified by univariate analysis as possible risk factors for ECCA death and only the earlier year of diagnosis was connected to conflicting reasons for death, though. The appropriate CIF curves (Fig. 1) and the OS layered in accordance with the aforementioned features (Fig. 2) showing that under age 65 years, earlier T stage, no distant lymph node metastases, no distant metastasis, earlier TNM stage, earlier tumor grading, surgical intervention, and radiation treatment were associated with greater OS.

**Table 1:** Overall survival rates and cumulative incidences of mortality among patients with advanced ECCA

Characteristic		Patients		Overall Survival rate(%)			*P*	Cancer-special mortality(%)			*P*	Non-cancer-special mortality(%)			*P*
		No.	%	1 year	2 year	3 year		1 year	2 year	3 year		1 year	2 year	3 year	
Total		403	100.0	31.3	12.0	7.0		58.7	74.2	78.0		10.0	13.8	15.0	
Age (years)	< 65	167	41.4	40.1	15.8	10.8	0.014	52.0	72.1	75.8	0.447	7.9	12.1	13.3	0.744
	≥65	236	58.6	25.1	9.4	4.3		63.4	75.7	79.6		11.5	14.9	16.2	
Sex	Female	192	47.6	29.4	8.9	4.7	0.130	61.7	78.5	81.7	0.143	8.9	12.6	13.6	0.594
	Male	211	52.4	33.1	14.9	9.0		55.9	70.3	74.7		11.0	14.8	16.3	
Race	Black	32	7.9	18.8	6.3	3.1	0.060	68.8	75.0	75.0	0.877	12.5	18.8	21.9	0.353
	White	371	92.1	32.4	12.5	7.3		57.8	74.2	78.3		9.8	13.3	14.4	
T	T0	5	1.2	20.0	20.0	20.0	0.041	80.0	80.0	80.0	0.232	0.0	0.0	0.0	0.795
	T1	91	22.6	17.8	5.6	3.3		71.1	77.7	80.0		11.1	16.7	16.7	
	T2	107	26.6	31.2	13.2	8.2		60.3	74.5	79.5		8.5	12.3	12.3	
	T3	92	22.8	37.5	18.7	11.0		57.1	71.4	74.7		5.5	9.9	14.3	
	T4	108	26.8	38.0	10.2	4.6		47.2	73.1	77.8		14.8	16.7	17.6	
N	N0	196	48.6	25.3	9.3	4.7	0.005	65.4	78.3	81.4	0.062	9.3	12.4	13.9	0.761
	N1	181	44.9	38.9	16.1	9.9		50.5	69.4	74.6		10.6	14.1	15.6	
	N2	26	6.5	23.1	3.9	3.9		65.4	76.9	76.9		11.5	19.2	19.2	
M	M0	196	48.6	47.4	20.9	12.6	< 0.001	39.8	63.3	70.5	< 0.001	12.8	15.8	16.9	0.353
	M1	207	51.4	15.8	3.5	1.5		76.9	84.8	85.3		7.4	11.8	13.3	
Stage	III	133	33.0	51.1	24.8	15.6	< 0.001	37.6	61.7	70.1	< 0.001	11.3	13.5	14.3	0.837
	IV	270	67.0	21.4	5.6	2.6		69.2	80.5	82.0		9.4	13.9	15.4	
Surgical	No	311	77.2	20.2	5.9	3.3	< 0.001	68.8	81.1	82.5	< 0.001	11.0	13.0	14.3	0.248
	Yes	92	22.8	68.5	32.6	19.3		25.0	51.1	63.3		6.5	16.3	17.4	
Grade	I	55	13.6	16.4	5.5	1.8	0.046	76.4	85.5	87.3	0.022	7.3	9.1	10.9	0.477
	II	179	44.4	34.8	13.7	8.0		55.0	71.5	76.6		10.2	14.8	15.3	
	III	165	40.9	32.7	12.1	7.1		56.4	73.3	76.5		10.9	14.5	16.4	
	IV	4	1.0	25.0	25.0	25.0		75.0	75.0	75.0		–	–	–	
Year of diagnosis	2000–2009	32	7.9	43.8	9.4	0.0	0.770	25.0	43.8	–	< 0.001	31.3	46.9	–	< 0.001
	2010–2017	371	92.1	30.2	12.3	7.6		61.6	76.9	81.0		8.1	10.9	11.4	
Radiation	No	294	73.0	31.0	12.4	7.9	< 0.001	59.4	74.9	78.3	0.507	9.6	12.7	13.7	0.359
	Yes	109	27.0	32.1	11.0	4.3		56.9	72.5	77.2		11.0	16.5	18.5	
Chemtherapy	No	223	55.3	28.7	9.1	5.5	0.150	60.8	77.7	80.4	0.198	10.5	13.2	14.1	0.592
	Yes	180	44.7	34.4	15.6	8.8		56.1	70.0	75.1		9.4	14.4	16.1	
Diagnostic Confirmation	Clinical diagnosis only	3	0.7	33.3	0.0	0.0	0.100	66.7	–	–	0.483	0.0	–	–	0.949
	Direct visualization without microscopic confirmation	1	0.2	0.0	0.0	0.0		–	–	–		–	–	–	
	Positive exfoliative cytology, no positive histology	86	21.3	23.3	5.8	3.5		65.1	79.1	81.4		11.6	15.1	15.1	
	Positive histology	294	73.0	33.7	13.8	7.8		57.0	72.8	77.0		9.3	13.4	15.1	
	Radiography without microscopic confirm	19	4.7	31.6	15.8	10.5		52.6	68.4	73.7		15.8	15.8	15.8

### Factor analysis for OS and CSS in univariate and multivariate form

**Fig. 3:**
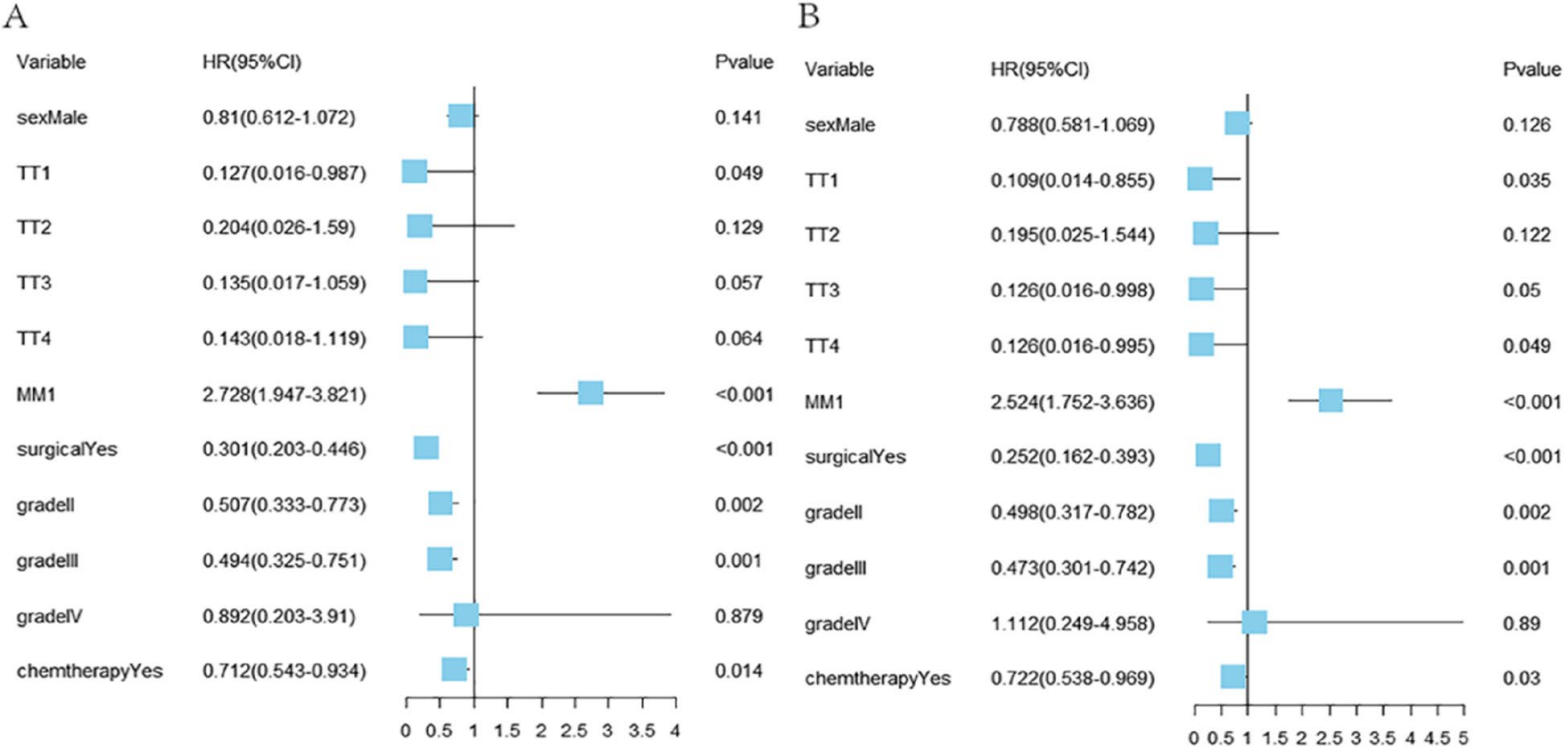
Multivariate analyses of survival in patients with advanced ECCA: **A** Overall survival; **B** Cancer-specific survival

Gender, race, T stage, N stage, distant metastases, grade, and surgical treatment were substantially linked with survival in the univariate COX analysis of OS, as shown in Table S2. Additionally, gender, T stage, lymph node metastasis, distant metastasis, TNM stage and surgical treatment were all found to be substantially linked with OS and CSS by univariate competing risk analysis. Although neither radiotherapy nor chemotherapy was significantly correlated with OS and CSS in the univariate analysis, they were frequently associated with prognosis in the clinical setting. Consequently, chemotherapy, radiation, and the relevant prognostic variables listed above were combined to create a multifactorial COX analysis. As Fig. 3 showed that the independent risk variables for OS and CSS were revealed to be T stage, distant metastasis, surgical intervention, grading, and chemotherapy treatment.

### The nomogram’s creation and validation

**Fig. 4:**
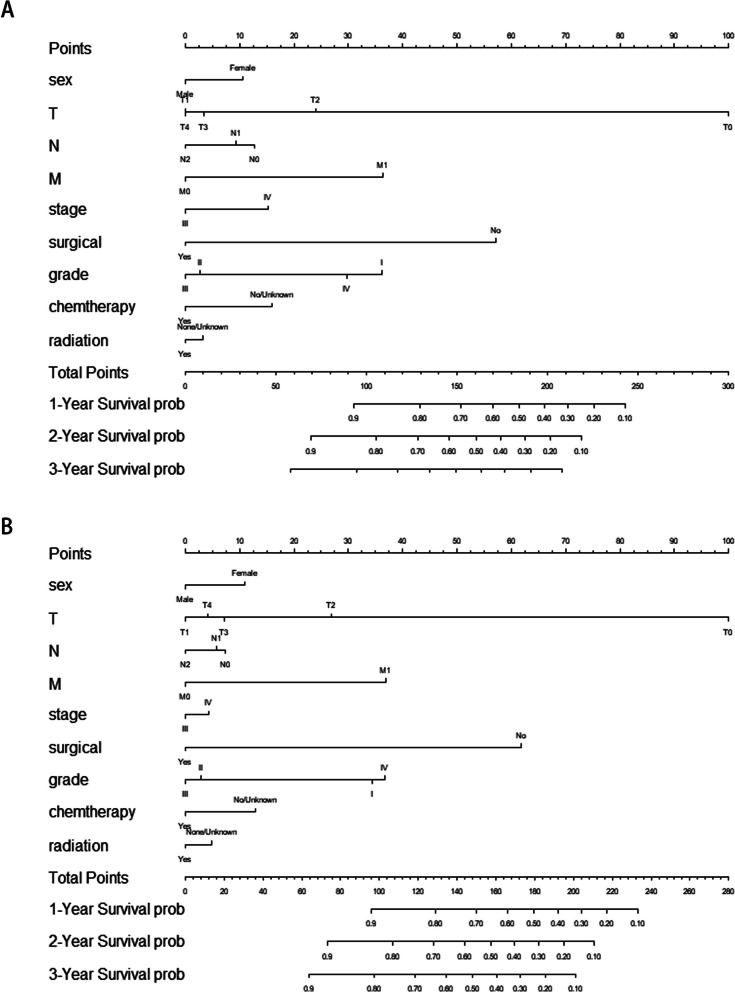
Nomograms predicting 1-, 2-, and 3-year OS (**A**) and CSS (**B**) of patients with advanced ECCA

**Fig. 5:**
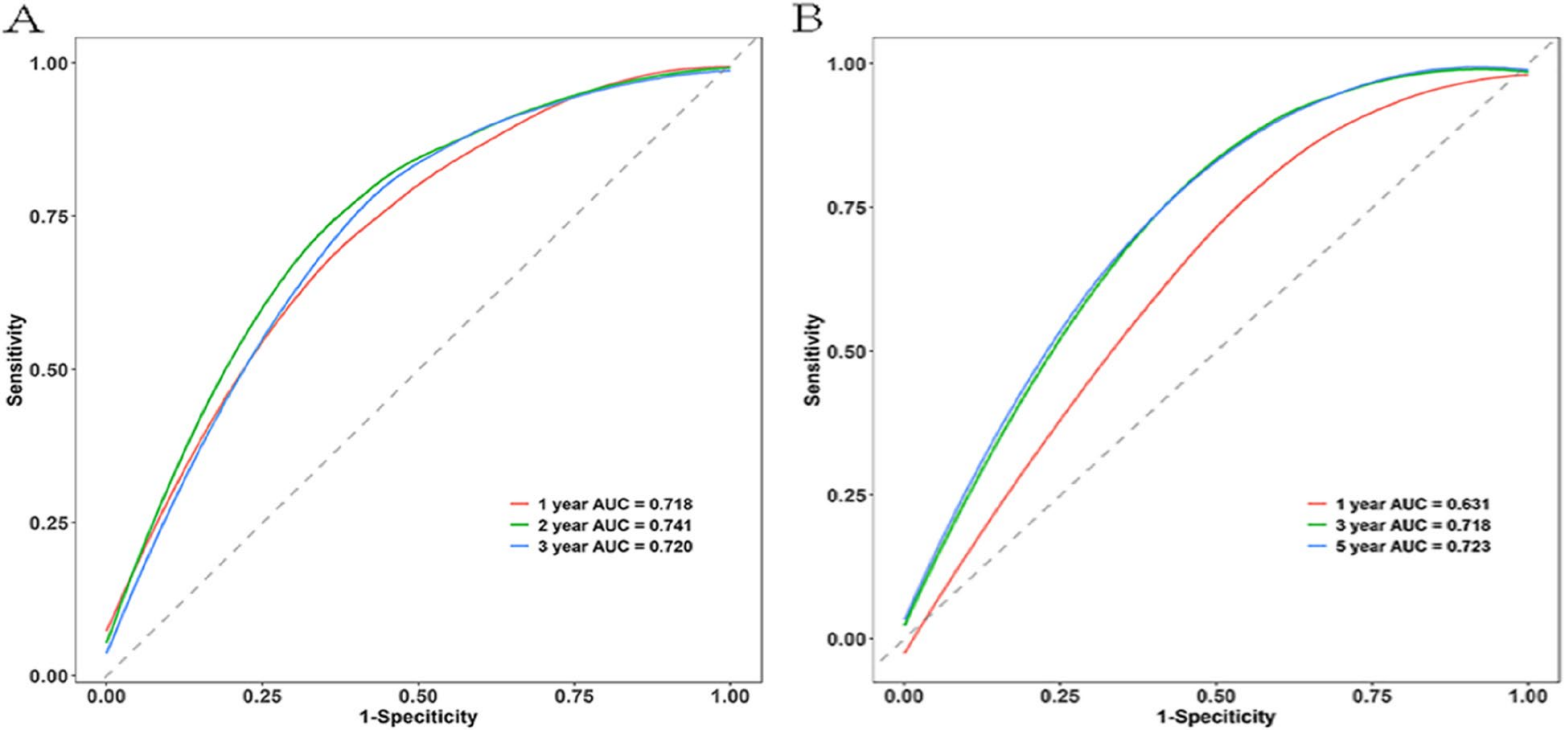
ROC curves for the nomogram for 1-, 2-, and 3-year OS prediction. **A** the training cohort; **B** the validation cohort

**Fig. 6:**
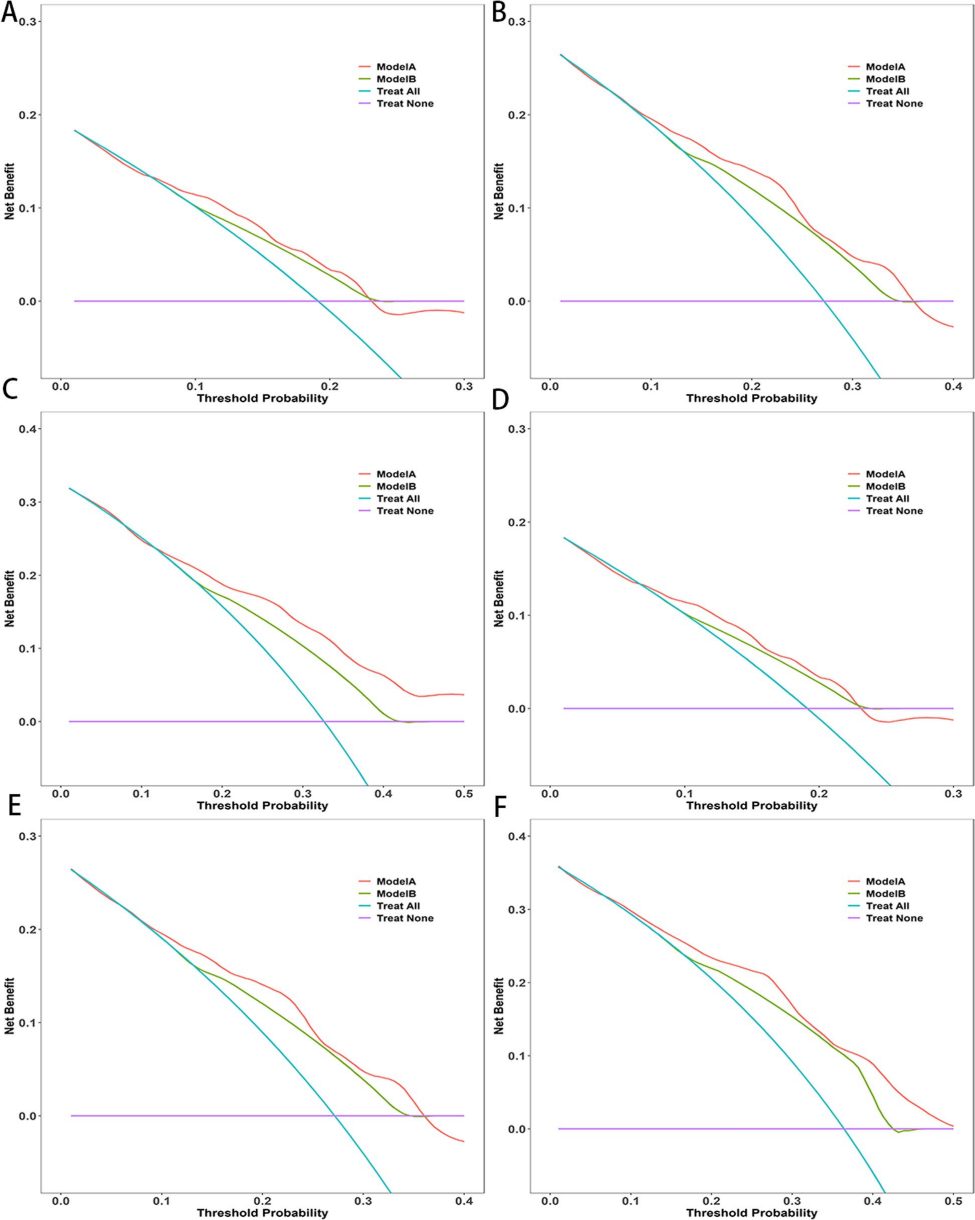
Decision curve analysis of 1-year, 2-year, and 3-year OS. **A**, **B**, **C** DCA curves of 1-year, 2-year, and 3-year OS in the training cohort; **D**, **E**, **F** DCA curves of 1-year, 2-year, and 3-year OS in the validation cohort; modle A,the prediction nomogram; modle B, the 7th AJCC stage

**Fig. 7:**
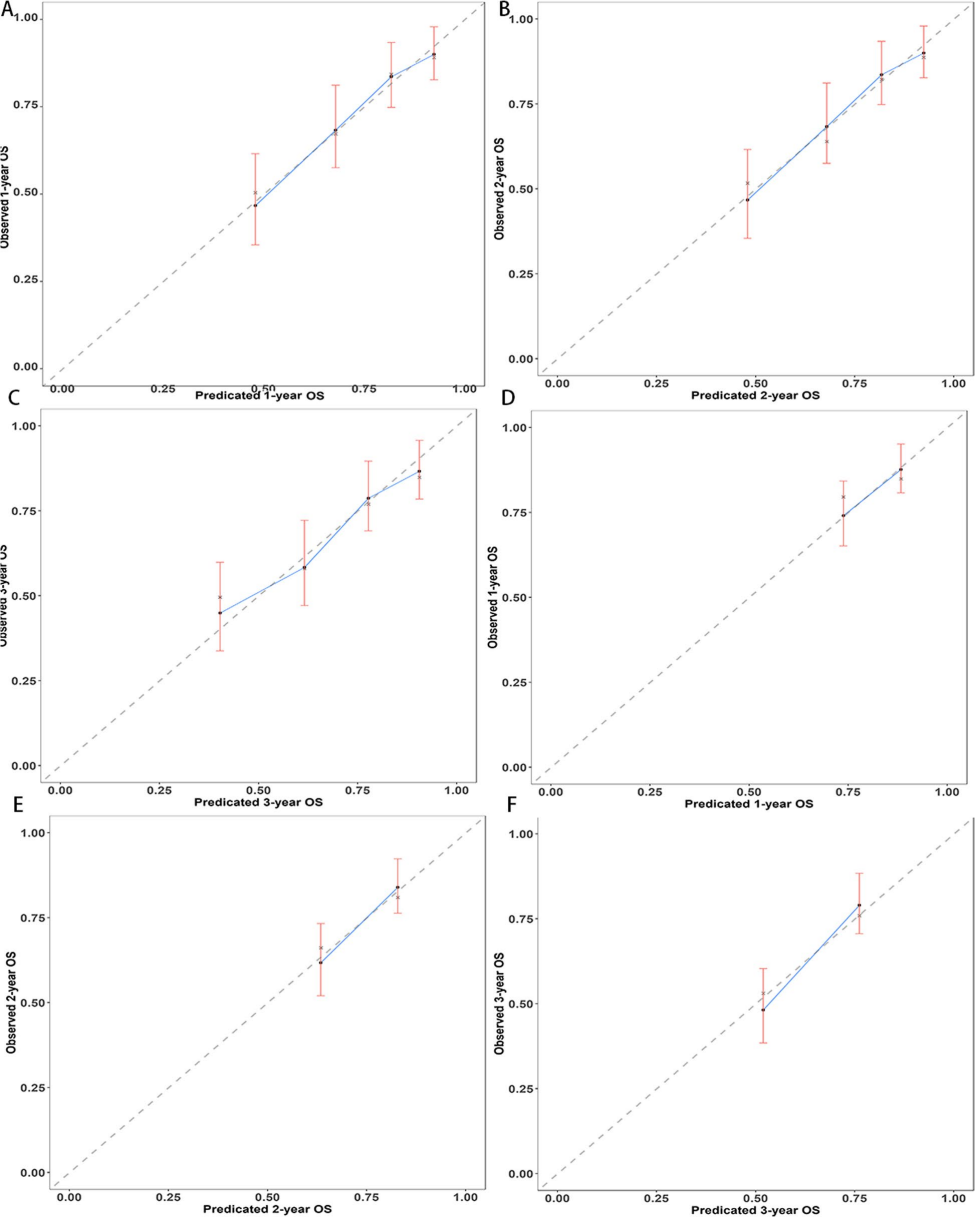
Calibration plots of 1-year, 2-year, and 3-year OS for advanced ECCA patients. **A**, **B**, **C** Calibration plots of 1-year, 2-year, and 3-year OS in the training cohort; **D**, **E**, **F** Calibration plots of 1-year, 2-year, and 3-year OS in the validiation cohort

As illustrated in Fig. 4, based on the results of the multifactor analysis the nomograms were developed utilizing the aforementioned predictors to anticipate OS and CSS at 1-, 2-, and 3-year. For the training cohort and validation cohort, respectively, the C-indexes with regards to nomograms used for predicting OS were 0.71 (95% CI: 0.68–0.75) and 0.65 (95% CI: 0.60–0.69). The nomograms for predicting CSS for the training and validation groups had C-indexes of 0.73 (95% CI: 0.69–0.76) and 0.68 (95% CI: 0.62–0.73), respectively. For OS the model’s strong clinical predictive ability was demonstrated by its 1-, 2-, and 3-year AUC values in the training cohort and validation cohort, which were 0.718, 0.720, 0.754 (Fig. 5A), and 0.631 0.718, 0.723 (Fig. 5B), respectively. The comparable ROC curves were shown in Fig. S1 for CSS. Further decision curve analysis (DCA) of OS and CSS revealed excellent potential for clinical application as well as nice positive net benefits in both the training and validation groups (Fig. 6, S2). The predicted and observed values of 1-, 2-, and 3-year were highly comparable in both the training and validation groups, as shown by the OS calibration curve (Fig. 7) and CSS calibration curve (Fig. S3), demonstrating the nomograms had great accuracy.

### Clinical value of nomograms based on AJCC staging compared with tumor staging

Using the C-index, NRI, ROC, and IDI, we assessed the precision of the nomograms and the 7th AJCC staging. The staged C-index for the 7th AJCC stage was much lower at 0.651 than the OS and CSS C-indices in the training cohort, which were 0.714 and 0.725, respectively. Similar to this, as showed in Table 2, the OS and CSS C-indices for the validating cohort were 0.647 along with 0.675, respectively, and the AJCC staging C-index was lower. For the 1-year, 2-year, and 3-year in the OS training cohort, the NRI was 0.56 (95% CI: 0.36–0.77), 0.55 (95% CI: 0.31–0.80), and 0.49 (95% CI: 0.24–0.72), respectively. The corresponding NRI of CSS 0.66 (95% CI: 0.43–0.88), 0.59 (95% CI: 0.35–0.77), and 0.49 (95% CI: 0.32–0.67) were found in the CSS training cohort, respectively. Additionally, the IDI was 0.12 (*p*<0.001), 0.14 (*p*<0.001), and 0.13 (*p*<0.001) for the 1-year, 2-year, and 3-year OS and the same conclusion was reached that the IDI for CSS was 0.13 (*p*<0.001), 0.14 (*p*<0.001), and 0.13 (*p*<0.001), separately. As evidence of the model’s potent predictive power, the areas under the ROC curves (AUCs) for the nomograms at 1-year, 2-year, and 3-year were 0.718, 0.741, and 0.720 for OS, which were higher than as opposed to areas for the AJCC staging method (Table 3). Similar to the previous example, it showed CSS nomograms also had a great prediction capacity, and its AUCs were much higher than those of the 7th AJCC stage (Table S3). Using DCA, the net advantages of nomograms and traditional clinical staging were evaluated.

**Table 2:** C-indexes for the nomograms and TNM stage systems in patients with advanced ECCA

C-index	Training cohort		Validation cohort	
	HR	95%CI	HR	95%CI
Overall survival	0.714	0.677–0.751	0.647	0.600–0.694
Cancer-specific survival	0.725	0.688–0.762	0.675	0.624–0.726
AJCC	0.651	0.612–0.690	0.591	0.548–0.634

**Table 3:** Comparison of the AUC values between the OS nomograms and TNM stage

AUC	Training cohort			Validation cohort		
	1-year	2-year	3-year	1-year	2-year	3-year
Nomogram	0.718	0.741	0.720	0.634	0.644	0.718
7th edition TNM stage	0.562	0.604	0.626	0.591	0.618	0.636

### Using the nomogram to create a tiered risk management system

**Fig. 8:**
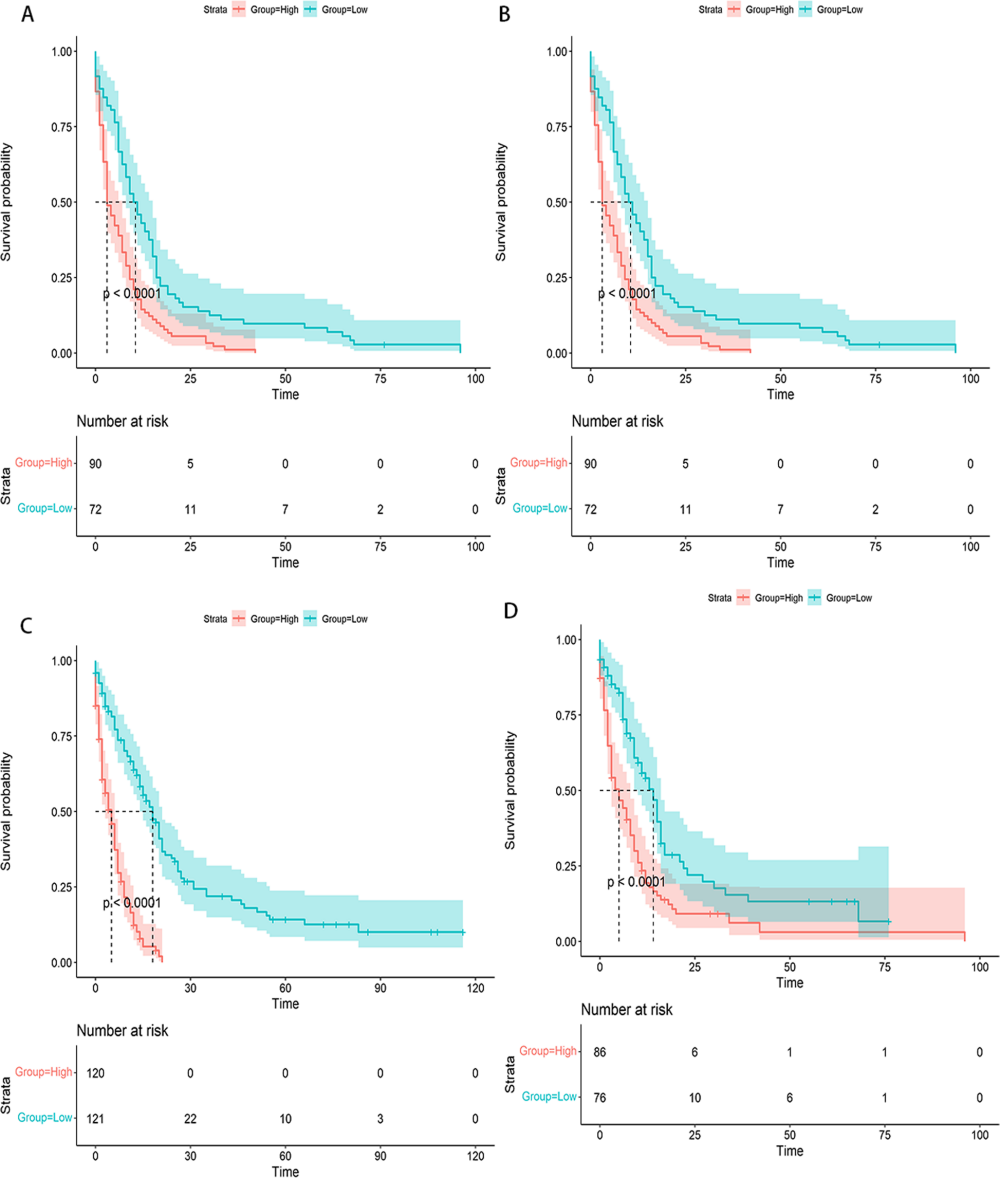
Kaplan–Meier OS and CSS curves of patients with advanced ECCA based on the new risk stratification system. **A**, **C** Kaplan–Meier curves of the raining cohorts; **B**, **D** Kaplan–Meier curves of the validation cohorts; **A**, **B** Kaplan–Meier OS curves; **C**, **D** Kaplan–Meier CSS curves

Patients with advanced ECCA were divided into high-risk and low-risk categories according to risk stratification that was done using the median of the cumulative scores derived from the nomogram scores as a threshold. The revised prognostic column plots then showed a substantial grading capacity according to Kaplan-Meier survival curve analysis (*p*<0.0001). In both the training and validation groups for OS and CSS, the low-risk group discovered a favorable prognosis (Fig. 8).

## Discussion

ECCA, as a rare epithelial malignancy, has been increasing in mortality and morbidity in recent years [21]. Previous studies of ECCA patients have shown that age, stage, surgery, and chemotherapy were associated with overall survival [22]. However, there are no studies evaluating prognostic events in patients with advanced ECCA and the competing risk factors were not analyzed. Consequently, we employed CSS in addition to OS, which does not account for competing risk factors, to assess factors that affect patient prognosis, removing the impact of competing causes of mortality. And the nomograms with excellent discrimination and calibration were constructed to predict OS and CSS in patients with advanced ECCA in this study for the first time. Furthermore, higher AUC values and C-indexes in patients with advanced ECCA both confirmed the superior discrimination of the nomograms compared with the 7th AJCC stage. By the time of follow-up, we found a 3-year cumulative mortality rate of 78.0 and 15.0% for CSS and non-cancer-specific mortality, respectively, indicating a large competitive relationship between them. Generally, a higher age is the main reason for a competitive relationship [23]. However, in the present study, increasing age was not significantly associated with OS in patients with advanced ECCA and was more irrelevant in CSS. This is contrary to the conclusions obtained from previous studies [24], may be related to the low overall survival rate of ECCA and the study population of advanced patients. Therefore, age may be temporarily excluded as a consideration when assessing whether patients with advanced ECCA have a favorable prognosis. Gender was a predictive factor in univariate analysis in the current study (OS:*P* = 0.018, CSS:*P* = 0.017), but it had no statistically significant impact with regard to any of the multifactorial variables (OS:*P* = 0.141, CSS:*P* = 0.126). However, investigations on other cancers revealed that gender was an independent prognostic factor and that males’ survival times were much shorter than females’. Both the study of Yu et al. study on elderly colonized patients [25] and the study of Wang et al. study on SCLC patients came to the same conclusions [26].

The survival of patients suffering from advanced ECCA is related to other predictors, such as the T stage and M stage, in the nomograms to predict OS as well as CSS. However, in both OS and CSS, the univariate analysis revealed that the N stage and TNM stage were the independent predictive indicators and not in the multifactorial analysis. This may be due to the small number of lymph node metastases in the current study, particularly N2, and the brief follow-up observation period. In addition, tumor grade, an inherent characteristic, was taken into account when assessing survival in patients with advanced ECCA. This study determined that grade was connected to both OS and CSS and was a distinct risk factor for patients who had advanced ECCA, which is consistent with other prior related studies. As according to Khan et al., patients with higher grades were more likely to have shorter survival time [22] and in other studies the same conclusion has been presented [27]. Additionally, this study included diagnostic techniques in the observed variables, but due to variations in subgroups, sample sizes, and study periods, there was no significant link with the prognosis of patients with advanced ECCA.

Radical surgical resection is the only way to treat bile duct cancer [28], achieving a negative margin R0 can dramatically improve patient survival. It has been indicated that lymph node status is one of the major prognostic factors after R0 resection for cholangiocarcinoma [29, 30], so local lymph node dissection with a clear scope of lymphatic resection is important to improve the overall survival rate of patients [31]. For patients who cannot be operated on or who need further care following surgery, adjuvant therapy is aslo crucial. Capecitabine is currently considered a conventional adjuvant chemotherapy agent [6], and fluorouracil and gemcitabine are also frequently used [32]. In this study, it was shown that patients can have a good prognosis after treatment with surgery or chemotherapy. The result is in line with the prior finding, as Greenberg et al. suggested that patients with ECCA had been demonstrated to experience significant prognostic advantages from adjuvant chemotherapy [33]. However, the present study obtained results contrary to previous studies that showed radiotherapy did not provide any benefit to patients with advanced ECCA. Although Razumilava et al. showed that a strong association between radiotherapy and the prognosis of ECCA patients [34]. And radiotherapy can support surgical treatment to assist patients experience larger advantages, according to Wang et al. [35].

AJCC staging is widely used to determine a patient’s prognosis for cancer with the disadvantage of not taking age, disease grading, and adjuvant therapy into account [7]. In this study using an extensive database, we carried out a risk assessment analysis and created nomograms to forecast the influencing factors impacting OS and CSS of patients with advanced ECCA for the first time. The nomogram facilitates the development of individualized treatment regimens by physicians, offers more precise prognostic predictions, and has been applied to the evaluation of a variety of malignancies such as colorectal cancer,osteosarcoma and lung cancer [36–38]. Prognostic nomogram establishment was carried out in ECCA patients by Fang et al. showed that nomograms are more predictive for patient survival than AJCC staging [23]. As in our study, in comparison to the 7th AJCC stage, the produced nomograms displayed higher time-dependent C-indexes and AUC values, demonstrating their modified discriminative ability to predict OS and CSS. It was also observed that the actual survival rate had a high agreement with the predicted raw survival rate of the nomograms based on the calibration plots, demonstrating the reliability of the nomograms. Moreover, the DCA results showed that the nomograms had more predictive power for survival compared to the AJCC stage and the results of IDI and NRI also supported the view that nomograms were reliable and accurate.

We divided the patients into groups with high and low risk in accordance with the general nomogram scores before doing the Kaplan-Meier survival analysis. According to the score information, patients in the group with a high risk showed considerably worse OS and CSS survival rates compared with those in the group with a low risk. In this study, to get a result that can be broadly applied in the current investigation, we used a substantial sample size. It can assist physicians in the treatment process to stratify risk according to the nomograms, to better assess the predictive characteristics of patients, and to take more aggressive treatment measures for high-risk patients, while overtreating low-risk patients can be avoided. In the future, receiving more specialized, personalized medical care will be possible.

One of the limitations of the study is that the normal clinical findings of CA19–9, CEA, and degree of vascular invasion were not collected in the SEER database, making it unable to assess these variables. The accuracy of determining the prognosis of patients with ECCA has been reported to be improved by including CA19–9 in the AJCC staging [39]. In addition, because this study was retrospective and did not obtain information on any specific surgery. It is not yet clear in analyzing the relationship between surgical modality and prognosis, and further research is needed. Meanwhile this study’s accuracy aslo needs to be further improved and a bigger sample size was required for the external validation examination in the future.

## Conclusion

In conclusion, we developed a new large-scale population-based staging system to estimate OS and CSS in patients with advanced ECCA. The nomogram provides precise information and aids clinicians in more thoroughly identifying potential risk factors and classifying patients into high- and low-risk categories because to its statistical foundation and reliable predictive power. As a result, doctors can utilize the model to direct patients down the right therapy courses, increasing individual survival. In the future, the large clinical trials and external validation will support our findings.

### Supplementary Information

**Additional file 1: Table S1.** Clinical characteristics of patients in the training cohort and validation cohort. **Table S2.** The results of univariate Cox regression analyses on variables for the prediction of OS and CSS. **Table S3.** Comparison of the AUC values between nomograms and TNM stage.

## Data Availability

The datasets generated and analyzed during the current study are available in the SEER database (https://seer.cancer.gov/).

## Abbreviations

HCC: Hepatocellular carcinoma
CCA: Cholangiocarcinoma
ICCA: Intrahepatic cholangiocarcinoma
ECCA: Extrahepatic cholangiocarcinoma
OS: Overall survival
CSS: Cancer-specific survival
SEER: Epidemiology and End Results
CIF: Cumulative incidence function
C-index: Concordance index
ROC: Operating characteristic curve
DCA: Decision curve analysis
NRI: Net reclassification index
IDI: Integrated discriminant improvement
AJCC: he American Joint Committee on Cancer
TNM: Tumor-Node-Metastasis
IQR: Interquartile range
