# Supplementary material for: Prognostic model for oversurvival and tumor-specific survival prediction in patients with advanced extrahepatic cholangiocarcinoma: a population-based analysis
Source: BMC Gastroenterol. 2023 Nov 30;23:422. doi: 10.1186/s12876-023-03017-6 (PMC10691049; doi:10.1186/s12876-023-03017-6)
Supplement: Supplementary file 1 — Additional file 1: Table S1. Clinical characteristics of patients in the training cohort and validation cohort. Table S2. The results of univariate Cox regression analyses on variables for the prediction of OS and CSS. Table S3. Comparison of the AUC values between nomograms and TNM stage. [file 12876_2023_3017_MOESM1_ESM.zip › Table S1.docx]

Table S1. Clinical characteristics of patients in the training cohort and validation cohort

| Variable | Overall (%) | Trainning cohort(%) | Validation cohort(%) | P value |
| --- | --- | --- | --- | --- |
|  | N=403 | N=241 | N=162 |  |
| age: |  |  |  | 0.939 |
| ＜65 | 167 (41.4%) | 99 (41.1%) | 68 (42.0%) |  |
| ≥65 | 236 (58.6%) | 142 (58.9%) | 94 (58.0%) |  |
| sex: |  |  |  | 0.174 |
| Female | 192 (47.6%) | 122 (50.6%) | 70 (43.2%) |  |
| Male | 211 (52.4%) | 119 (49.4%) | 92 (56.8%) |  |
| race: |  |  |  | 0.539 |
| Black | 32 (7.94%) | 17 (7.05%) | 15 (9.26%) |  |
| White | 371 (92.1%) | 224 (92.9%) | 147 (90.7%) |  |
| T: |  |  |  | 0.317 |
| T0 | 5 (1.24%) | 1 (0.41%) | 4 (2.47%) |  |
| T1 | 91 (22.6%) | 50 (20.7%) | 41 (25.3%) |  |
| T2 | 107 (26.6%) | 68 (28.2%) | 39 (24.1%) |  |
| T3 | 92 (22.8%) | 56 (23.2%) | 36 (22.2%) |  |
| T4 | 108 (26.8%) | 66 (27.4%) | 42 (25.9%) |  |
| N: |  |  |  | 0.901 |
| N0 | 196 (48.6%) | 115 (47.7%) | 81 (50.0%) |  |
| N1 | 181 (44.9%) | 110 (45.6%) | 71 (43.8%) |  |
| N2 | 26 (6.45%) | 16 (6.64%) | 10 (6.17%) |  |
| M: |  |  |  | 0.092 |
| M0 | 196 (48.6%) | 126 (52.3%) | 70 (43.2%) |  |
| M1 | 207 (51.4%) | 115 (47.7%) | 92 (56.8%) |  |
| stage: |  |  |  | 0.522 |
| III | 133 (33.0%) | 83 (34.4%) | 50 (30.9%) |  |
| IV | 270 (67.0%) | 158 (65.6%) | 112 (69.1%) |  |
| surgical: |  |  |  | 0.720 |
| No | 311 (77.2%) | 184 (76.3%) | 127 (78.4%) |  |
| Yes | 92 (22.8%) | 57 (23.7%) | 35 (21.6%) |  |
| grade: |  |  |  | 0.868 |
| I | 55 (13.6%) | 34 (14.1%) | 21 (13.0%) |  |
| II | 179 (44.4%) | 104 (43.2%) | 75 (46.3%) |  |
| III | 165 (40.9%) | 101 (41.9%) | 64 (39.5%) |  |
| IV | 4 (0.99%) | 2 (0.83%) | 2 (1.23%) |  |
| year.of.diagnosis: |  |  |  | 0.891 |
| 2000-2009 | 32 (7.94%) | 20 (8.30%) | 12 (7.41%) |  |
| 2010-2017 | 371 (92.1%) | 221 (91.7%) | 150 (92.6%) |  |
| radiation: |  |  |  | 0.448 |
| None/Unknown | 294 (73.0%) | 172 (71.4%) | 122 (75.3%) |  |
| Yes | 109 (27.0%) | 69 (28.6%) | 40 (24.7%) |  |
| chemtherapy: |  |  |  | 0.108 |
| No/Unknown | 223 (55.3%) | 125 (51.9%) | 98 (60.5%) |  |
| Yes | 180 (44.7%) | 116 (48.1%) | 64 (39.5%) |  |
| Diagnostic.Confirmation: |  |  |  | 0.456 |
| Clinical diagnosis only | 3 (0.74%) | 3 (1.24%) | 0 (0.00%) |  |
| Direct visualization without microscopic confirmation | 1 (0.25%) | 1 (0.41%) | 0 (0.00%) |  |
| Positive exfoliative cytology, no positive histology | 86 (21.3%) | 53 (22.0%) | 33 (20.4%) |  |
| Positive histology | 294 (73.0%) | 175 (72.6%) | 119 (73.5%) |  |
| Radiography without microscopic confirm | 19 (4.71%) | 9 (3.73%) | 10 (6.17%) |  |
| time | 7.00 [2.00;15.0] | 7.00 [2.00;15.0] | 7.00 [2.00;13.8] | 0.446 |
